# Temporal Progression of Fatty Acids in Preterm and Term Human Milk of Mothers from Switzerland

**DOI:** 10.3390/nu11010112

**Published:** 2019-01-08

**Authors:** Sagar K. Thakkar, Carlos Antonio De Castro, Lydie Beauport, Jean-François Tolsa, Céline J. Fischer Fumeaux, Michael Affolter, Francesca Giuffrida

**Affiliations:** 1 Nestlé Institute of Health Sciences, Nestlé Research, Lausanne 1000, Switzerland; Sagar.Thakkar@rd.nestle.com; 2 Clinical Development Unit, Nestlé Research Asia, Singapore 138567, Singapore; CarlosAntonio.DeCastro@rdsg.nestle.com; 3 Clinic of Neonatology, Department Woman Mother Child, University Hospital of Lausanne, Lausanne 1011, Switzerland; Lydie.Beauport@chuv.ch (L.B.); Jean-Francois.Tolsa@chuv.ch (J.-F.T.); Celine-Julie.Fischer@chuv.ch (C.J.F.F.); 4 Nestlé Institute of Food Safety & Analytical Science, Nestlé Research, Lausanne 1000, Switzerland; Michael.Affolter@rdls.nestle.com

**Keywords:** human milk, preterm, term, infants, lipids, fatty acids, human milk fortification, docosahexaenoic acid (DHA), mothers’ own milk, donor human milk, arachidonic acid (ARA), eicosapentaenoic acid (EPA)

## Abstract

We longitudinally compared fatty acids (FA) from human milk (HM) of mothers delivering term and preterm infants. HM was collected for 4 months postpartum at 12 time points for preterm and for 2 months postpartum at 8 time points for term group. Samples were collected from the first feed of the morning, and single breast was fully expressed. FA were analyzed by gas chromatography coupled with flame ionization detector. Oleic, palmitic and linoleic acids were the most abundant FA across lactation and in both groups. Preterm colostrum contained significantly (*p* < 0.05) higher 8:0, 10:0, 12:0, sum medium chain fatty acids (MCFA), 18:3 n-3 FA compared to term counterparts. Preterm mature milk contained significantly higher 12:0, 14:0, 18:2 n-6, sum saturated fatty acids (SFA), and sum MCFA. We did not observe any significant differences between the preterm and term groups for docosahexaenoic acid, arachidonic acid and eicosapentaenoic acid at any stage of lactation. Overall, preterm milk was higher for SFA with a major contribution from MCFA and higher in 18:2 n-6. These observational differences needs to be studied further for their implications on preterm developmental outcomes and on fortification strategies of either mothers’ own milk or donor human milk.

## 1. Introduction

Given the documented short- and long-term advantages of breastfeeding for both the mother and the infant, it is no surprise that breastfeeding and human milk (HM) feeding are considered as normative standards by health care professionals and organizations such as the World Health Organization (WHO), American Academy of Pediatrics and European Commission [1]. WHO recommends exclusive breastfeeding for the first six months of infant life followed by introduction of complementary foods and continued breastfeeding for up to two years of life, and even beyond until mutually agreeable by the mother-infant dyad [2]. Indeed, it has been stated that HM feeding during this early stage of life is able to meet the nutritional demands of not only growth and development, but also imparts the immune factors and protects from later in life metabolic abnormalities of apparently healthy term infants [3]. For preterm infants, HM confers additional benefits, while reducing morbidity and mortality and enhancing neurodevelopment of this vulnerable population [4]. However, feeding a preterm infant requires special considerations to meet the nutritional demands to allow for mimicking growth that would otherwise take place in-utero.

Understanding the roles of nutrition in general and of lipids in particular, developmental outcomes of preterm infants has been a subject of much research in an effort to define their nutritional needs [5,6,7,8,9]. Since HM feeding is well tolerated and reduces the risk of co-morbidities such as necrotizing enterocolitis and sepsis in preterm infants, it is important that feeding HM to preterm infants has become the primary choice of nutritional source [10]. Furthermore, strategies have also been developed to supplement mothers’ own milk (MOM) with either multi-nutrient or single nutrient human milk fortifiers (HMF) based on bed-side analyses of MOM [11]. Nevertheless, the bedside analyses of MOM has been focused on macronutrients (total proteins, total lipids, total lactose and calculated total energy) often using mid-infrared spectroscopic methods and not on their detailed profile or other micronutrients that may also contribute to growth and development [12].

Lipids in unfortified HM/MOM provide approximately 50% of the total energy to its consumers and the majority (90–95%) of lipids are present in the form of triacylglycerol (TAG), a glycerol molecule bound to three fatty acids (FA). These FA range from medium chain to very long chain and may be saturated, mono-unsaturated or polyunsaturated. Additionally, essential FA as well as non-nutritive bioactive FA are also part of the FA pool supplied via HM. Conventional data suggests that FA in HM may be modified by the maternal dietary or nutritional supplement intake but not the quantity or concentration of total lipids [13]. The concentration of HM lipids increases with advancing stages of lactation [14,15,16]. In fact, HM composition may be impacted by a multitude of factors ranging from maternal to infant parameters and even including the physiological and behavioral aspects observed in the mother-infant dyad. These parameters have been recently reviewed and summarized by Fields and colleagues [17]. Certainly one of those parameters that may influence the HM composition is the gestational age at the birth of infants. Mothers of preterm infants have a higher risk of delayed onset of lactogenesis II and potentially the mammary tissues may not be developed to the extent of their term mother counterparts [18]. Undoubtedly, more research is needed in this area to understand the impact of delayed milk production on the mother and the infant.

A handful of reports already exists on comparison of HM from mothers of term and preterm infants. However, they have either focused on selective FA such as arachidonic acid (ARA) or docosahexaenoic acid (DHA) [19,20], or focused only on transitional and mature milk [21]. Therefore, in this study we aimed to explore and compare the composition of FA in HM produced by mothers delivering a preterm infant to that of a term infant from colostrum, transitional and mature milk.

## 2. Materials and Methods

### 2.1. Ethical and Legal Considerations

This study was conducted according to the guidelines of the Declaration of Helsinki. The study protocol with all procedures involving human subjects was approved by the Ethical Board (Commission cantonal d’éthique de la recherché sur l’être humain) of the Canton de Vaud, Switzerland (Protocol 69/13, clinical study 11.39.NRC; April 9, 2013). Written consent was obtained from all participating subjects of the study. The study was registered at ClinicalTrials.gov with the identifier NCT02052245.

### 2.2. Study Settings and Subjects

The study was conducted between October 2013 and July 2014 at the neonatal intensive care unit (NICU) of the University Hospital (CHUV) in Lausanne, Switzerland. A longitudinal HM sampling from lactating mothers was performed for HM characterization. Subjects were recruited at the hospital within 2–3 days after giving birth (preterm and term births) by a single dedicated lactation nurse who managed all interactions with subjects from start to finish of the study. A total of 61 mothers were recruited for the study, out of which 27 had preterm deliveries and 34 had term deliveries.

### 2.3. Inclusion and Exclusion Criteria

Eligibility criteria for this study included women older than 18 years of age giving (1) preterm birth (between gestational ages 28 0/7 and 32 6/7 weeks) or (2) term birth (>37 0/7 and not above 41 6/7 weeks) with mothers’ intention to exclusively or partially breastfeed at least until 4 month post-partum. Exclusion criteria included gestational and pre-gestational diabetes (type I or II), alcohol or illicit drug consumption and insufficient skills to understand study questionnaire. Availability of refrigerator/freezer at home for storage of collected human milk samples was required.

### 2.4. Data Collection

After the subjects were enrolled and their signature obtained on the informed consent form, the following information was collected for (a) mothers: age, height, weight before pregnancy and at delivery, and (b) for the infants: date of birth, sex, gestational age, delivery method, weight, and head circumference and sibling related data. The dedicated study nurse conducted data collection in face-to-face interviews with the subjects.

### 2.5. Human Milk Sampling, Handling, and Storage

HM from mothers of preterm infants were sampled weekly for eight weeks post-partum and then once every two weeks for eight more weeks totaling 12 longitudinal samples. HM from mothers of term infants were sampled weekly for eight weeks post-partum totaling eight longitudinal samples. Various aspects of HM sampling were standardized for all subjects. Milk was collected between 06h00 and 12h00 using an electric breast pump (Symphony^®^, Medela, Baar, Switzerland) allowing the mothers flexibility to express at home or at the NICU. The side of the breast selected by the mother was kept the same during the entire study and the mothers were requested to empty the breast in the previous feed or the pumping session. Single full breast was sampled and an aliquot of 10 mL HM for each time point (or 1–3 mL for the first two sampling time points in the preterm group) was reserved for biochemical characterization. The remainder of the HM was returned to the mother for feeding to the infant at a later time point, if so required. Each sample was transferred to freezing tubes, labelled with subject number and collection information, stored at −18 °C in the home freezer, transferred to the hospital (storage at −80 °C) and then shipped to the Nestlé Research Centre (Lausanne, Switzerland) where it was stored at −80 °C until analysis. The frozen HM samples were thawed once for aliquoting into 15 individual small volume fractions (min 0.2 mL to max 2 mL) in separate polypropylene Eppendorf tubes dedicated to the different analyses. The aliquoting approach was implemented to avoid repeated thawing-freezing cycles and to adapt the required volumes to the specific needs of the individual analytical methods.

### 2.6. Quantification of Total Lipids in Human Milk

Total lipid content was measured in HM samples by a human milk analyzer (HMA). The device employed for analyses was a HMA generation 3 (Miris AB, Uppsala, Sweden) using the XMA-SW software version 2.87 (Miris AB, Uppsala, Sweden). This HMA is based on semi-solid middle infrared (MIR) transmission spectroscopy. The wavebands used are specific for the functional carbonyl groups (5.7 µm) for fat determination, amide groups (6.5 µm) for protein determination, and hydroxyl groups (9.6 µm) for carbohydrate determination. Prior to analysis, a daily calibration check was performed using the calibration solution provided by the supplier. All samples were homogenized for 3 × 10 s using the MIRIS sonicator (MIRIS AB, Uppsala, Sweden) as recommended by MIRIS and were kept in a water bath at 40 °C prior to measurement. Homogenized samples (1 mL) were injected into the flow cell and measured within a minute. Once the analysis was completed, the built-in cell and all lines were rinsed with deionized water. After five milk samples, the system was cleaned with the recommended MIRIS detergent. An in-house control sample as well as a calibration standard provided by the manufacturer were analyzed after every tenth measurement for quality control purposes.

### 2.7. Direct Method Procedure to Prepare Fatty Acid Methyl Esters (FAME) from Human Milk

Fatty acids were quantified in HM as described by Cruz-Hernandez et al. [22]. Acid FAME were prepared using HCl/Methanol (3N) as a catalyst. The methylation procedure was as follows: In a 15 mL test tube equipped with Teflon-lined screw caps, 250 µL of HM was added followed by 300 µL of internal standard FAME 11:0 and 300 µL of internal standard TAG 13:0, 2 mL of methanol, 2 mL of methanol/HCL (3N) and 1 mL of *n*-hexane. Test tubes were firmly capped and shaken vigorously and heated at 100 °C for 60 min, with occasional shaking. Care was taken to fit the cap tightly with cap liner to avoid leaks when tubes are heated at 100 °C. After cooling down to room temperature, 2 mL water was added and shaken vigorously for centrifugation at 1200× *g* for 5 min followed by transfer of the upper phase (hexane) into gas chromatography vials. Analyses were performed on a 7890A gas-chromatograph (Agilent Technologies, Palo Alto, CA, USA) equipped with a fused-silica CP-Sil 88 capillary column (100% cyanopropylpolysiloxane; 100 m, 0.25 mm id, 0.25 µm film thickness; Agilent, Palo Alto, CA, USA) have been used with a split injector (1:25 ratio) heated at 250 °C and a flame-ionization detector operated at 300 °C. Oven temperature programming used was 60 °C isothermal for 5 min, increased to 165 °C at 15 °C/min, isothermal for 1 min at this temperature, and then increased to 195 °C at 2 °C/min and held isothermal for 14 min and then increased to 215 °C at 5 °C/min and held isothermal for 8 min at 215 °C. Hydrogen was used as carrier gas under constant flow mode at 1.5 mL/min.

### 2.8. Statistics

The scarcity of quantitative data on the fatty acid content in preterm HM precluded a power calculation in this exploratory study. Sample size was initially set at *n* = 20 subjects per group (preterm and term groups), according to the estimated recruitment feasibility at the study center within a one year period. The longitudinal evolution of fatty acid content was compared in preterm and term HM postpartum age (categorized by lactation stages: colostrum (≤1 week postpartum), transitional (>1 week and ≤2 weeks post-partum) and mature (>2 weeks and ≤16 weeks)). No aggregation was done for observations from the same participant within each lactation stage (i.e., colostrum has 1 observation, transitional milk has 1 observation and mature milk has 4 observations per participant). Mixed linear models were used to estimate the differences between preterm and term HM. The models used age (colostrum, transitional milk and mature milk stages), term/preterm birth status, and interaction between age and term/preterm status and delivery mode. Within subject variability was accounted by declaring the subject ID as a random effect. Logarithmic transformation was applied to the FA as they are generally skewed. Only the ratios (n-6 to n-3 ratio and ARA to DHA ratio) were assumed to be normally distributed, therefore no transformation was applied. Contrast estimates of the model were calculated by comparing preterm and term HM groups at each time point. No imputation method was applied for missing data (both in between visits and loss to follow up) as the method used does not require a complete data set. A conventional 2-sided 5% error rate was used without adjusting for multiplicity. A similar analysis was done separating term and preterm infants but looking at the age differences (colostrum vs. transitional milk, transitional milk vs. mature milk and colostrum vs. mature milk). Statistical analyses were done with SAS 9.3 (SAS Institute Inc., Cary, NC, USA) and R 3.2.1. (R Foundation, Vienna, Austria) Differences were considered statistically significant when *p* values were <0.05.

## 3. Results

### 3.1. Subject Characteristics

**Figure 1:**
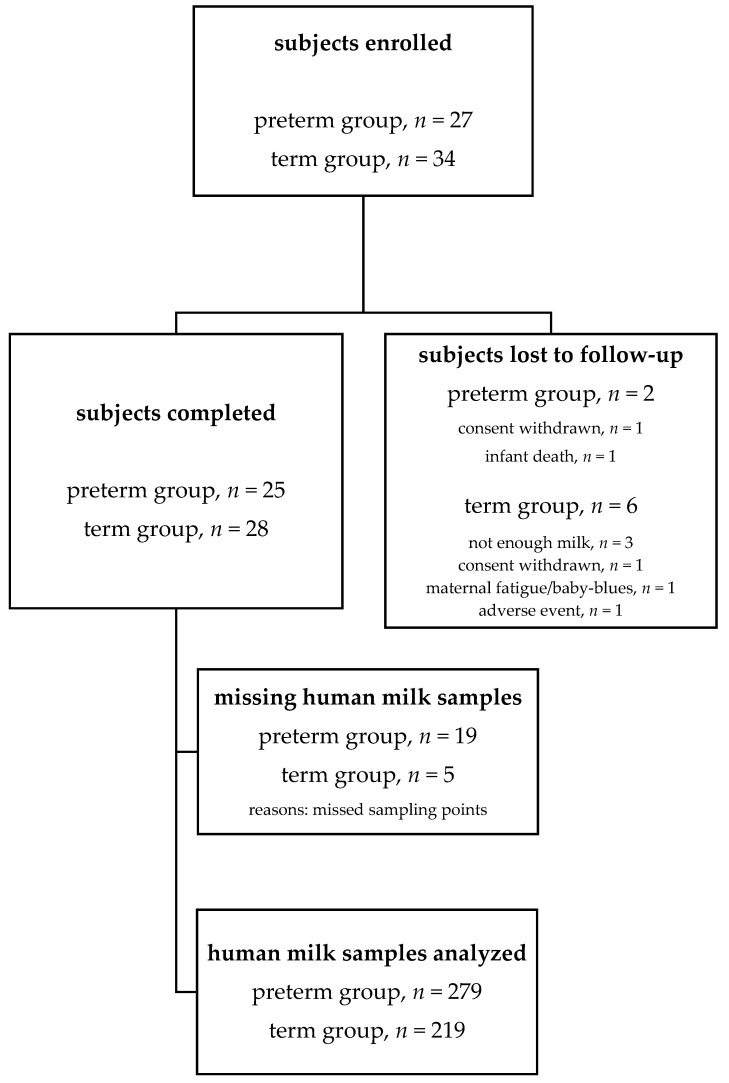
Study flow chart.

This study included convenient sampling of 27 mothers who delivered 33 preterm infants and 34 mothers who delivered 34 term infants at CHUV neonatal unit in Lausanne, Switzerland. Multiple deliveries (twins) were frequent (36%) in the preterm group, but absent in the term group. Figure 1 displays the study flow chart. Two out of 27 (7.4%) preterm infant mothers and 6 out of 34 (17.6%) term infant mothers were lost to follow-up. No adverse events were reported along the study period. In total, 498 HM samples, 279 from preterm and 219 from full term infant mothers, were available for fatty acid analyses. Table 1 reports mother, infant demographic, and baseline anthropometric data. Maternal characteristics were comparable among groups. Caesarean delivery was more frequent in the preterm group. Preterm and full term infants significantly differed in all parameters except for gender distribution.

### 3.2. Total Lipids in Preterm and Term Human Milk

**Figure 2:**
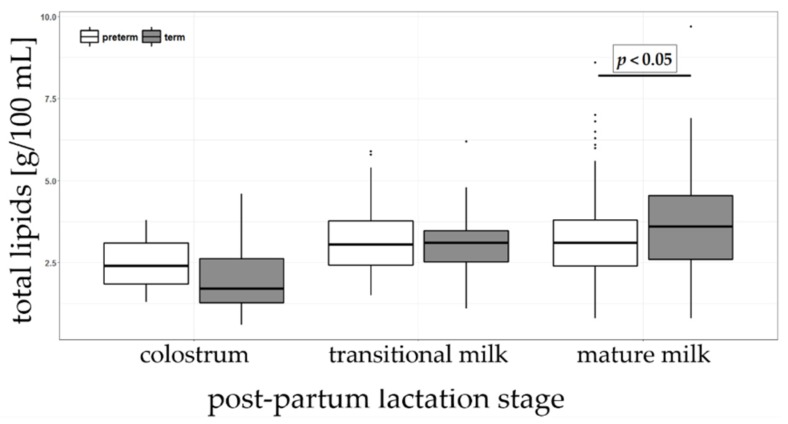
Total lipids content (g/100 mL) in colostrum, transitional and mature milk in preterm and term groups.

Total lipids was measured by MIRIS^®^ HMA as previously described by Giuffrida et al. [23] and results are listed in Table 2. Total lipids content increased from colostrum (2.4 and 1.7 g/100 mL preterm and term HM, respectively) to mature milk (3.1 and 3.6 g/100 mL in preterm and term HM, respectively). Significant differences in total lipid content were observed between preterm and term groups but only in mature milk (Figure 2).

### 3.3. Fatty Acids in Preterm and Term Human Milk

FA were measured by gas chromatography—flame ionization detection (GC-FID), and the results are enumerated in Table 2.

**Figure 3:**
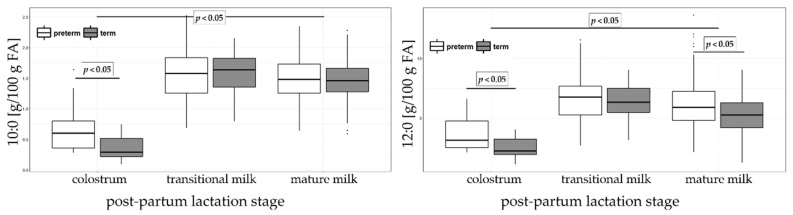
Capric (10:0, left panel) and lauric (12:0, right panel) acids in colostrum, transitional and mature milk in preterm and term groups. The results are expressed as percentages (g/100 g sum of FA).

The sum of saturated fatty acid (SFA) content increased significantly from colostrum (43.49 and 42.03% sum of FA in preterm and term HM, respectively) to mature milk (45.88 and 43.86% sum of FA in preterm and term HM, respectively). The sum of SFA content was significantly higher in preterm than in term mature milk. Palmitic acid (16:0) was the most abundant SFA and overall second most abundant FA in preterm and term HM in this study. In spite of increases in total SFA, palmitic acid content significantly decreased from colostrum (24.02 and 25.68% sum of FA in preterm and term HM, respectively) to mature milk (23.10 and 23.29% sum of FA in preterm and term HM, respectively). Stearic acid (18:0) increased significantly only in preterm milk (6.30% in colostrum and 7.03% in mature milk). Furthermore, short (SC; 8:0) and medium chain (MC; 10:0 and 12:0) FA content was significantly low in colostrum (3.95 and 2.51% sum of FA in preterm and term HM, respectively) compared to transitional (8.52 and 7.93% sum of FA in preterm and term HM, respectively) and mature milk (7.63 and 6.94% sum of FA in preterm and term HM, respectively). Significant differences were observed between preterm and term colostrum for caprylic (8:0), capric (10:0) and lauric (12:0) being higher in preterm than in term. In mature HM, lauric and myristic (14:0) acids were significantly higher in preterm than in term. Figure 3 shows capric and lauric acid concentration in colostrum, transitional and mature milk and differences between preterm and term. Arachidic acid (20:0) was lower in preterm milk but only for the colostrum stage of lactation.

**Figure 4:**
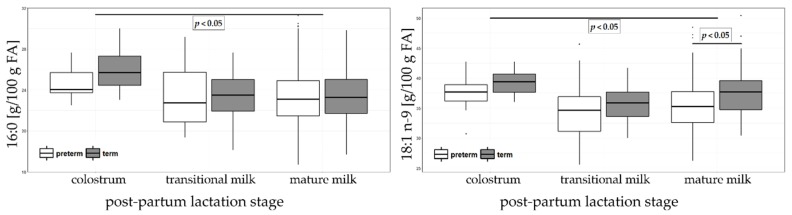
Palmitic (16:0, left panel) and oleic (18:1 n-9, right panel) acids in colostrum, transitional and mature milk in preterm and term. The results are expressed as percentages (g/100 g sum of FA).

The sum of MUFA in HM decreased significantly from colostrum (44.59 and 46.51% of sum of FA in preterm and term HM, respectively) to mature milk (40.44 and 43.84% of sum of FA in preterm and term HM, respectively). Oleic acid (18:1 n-9), the most abundant FA, decreased significantly along the lactation (from 37.64 to 35.22 % of total FA in preterm HM and from 39.36 to 37.67% of total FA in term HM) (Figure 4). Other MUFA (i.e., 18:1 n-7, 20:1 n-9, 22:1 n-9 and 24:1 n-9) also decreased significantly over lactation. The only exception was 16:1 n-7 for which no significant trend was observed. The total MUFA content was significantly higher in term than in preterm mature milk. However, significant differences were observed between preterm and term colostrum with 22:1 n-9 and 24:1 n-9 being significantly lower in preterm and in mature milk for 18:1 n-9 being significantly lower in preterm HM.

**Figure 5:**
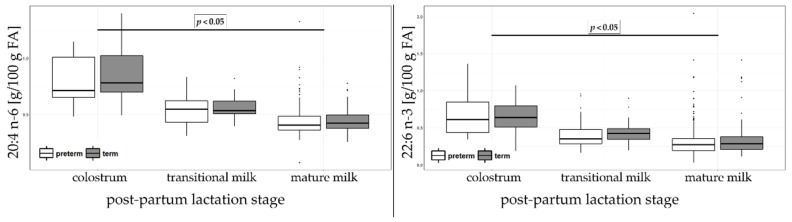
Arachidonic acid (ARA) (20:4 n-6, top panel) and docosahexaenoic acid (DHA) (22:6 n-3, bottom panel) acids in colostrum, transitional and mature milk in preterm and term. The results are expressed as percentages (g/100 g sum of FA).

Among poly-unsaturated fatty acids (PUFA), n-6 linoleic acid (LA; 18:2 n-6) was the most abundant FA and it increased significantly from colostrum (9.61 and 7.92% sum of FA in preterm and term HM, respectively) to mature milk (10.21 and 9.35% sum of FA in preterm and term HM, respectively). ARA (20:4 n-6) content decreased significantly from colostrum to mature milk in both preterm and term HM. No significant differences on PUFA n-6 content were observed between preterm and term in colostrum, transitional milk and mature milk (Figure 5). Among PUFA, n-3 alpha-linolenic acid (ALA; 18:3 n-3) was the most abundant FA and it increased significantly in term from colostrum (0.51% of total FA) to mature milk (0.74% of total FA) but it was stable at about 0.7% of sum of FA in preterm group. DHA (22:6 n-3) decreased significantly over the lactation period from 0.6% in colostrum to 0.3% of total FA in mature milk, in both preterm and term HM. Eicosapentaenoic acid (EPA; 20:5 n-3) was present in minute quantities (0.07% of the sum of FA in colostrum, transitional and mature milk). Between preterm and term, the only significant differences were observed for ALA in colostrum, being higher in preterm than in term HM.

## 4. Discussion

There are different classes of fatty acids (FA) in HM with putative biological functions. Most widely studied are long chain polyunsaturated FA (LCPUFA) with potential roles in the development of visual and cognitive functions in early life [24,25]. HM also contains essential FA, such as linoleic (18:2 n-6) and alpha-linolenic (18:3 n-3) acid that must be supplied orally as *de novo* synthesis is low to non-existent [7]. Furthermore, there is also the presence of short to medium chain and saturated FA (SFA) along with monounsaturated FA (MUFA). In this study, we longitudinally characterized total lipids and FA from milk of mothers who delivered either preterm or term infants.

Total lipid content increased from colostrum to mature milk in agreement with multiple previous reports [16,26,27,28,29,30,31,32,33]. However, our observation that term HM had higher lipids than preterm milk when the milk was mature is not corroborated by a systematic review and meta-analysis of Gidrewicz and Fenton [34]. This could be due to inclusion of multiple studies in meta-analysis with varied sampling and analytical procedures as compared to our study.

The saturated FA we characterized in this study ranges from short chain FA (8:0), medium chain FA (10:0, 12:0, 14:0), to long chain FA (16:0, 18:0, 20:0) and very long chain FA (24:0). Overall, the sum of all SFA in colostrum was lower than transitional and mature milk for both preterm and term groups. In colostrum, short and medium chain FA were higher in preterm than in term HM, in agreement with previous works [35,36]. Benefits of medium chain FA in preterm infant nutrition has been a topic of research for past few decades. It has been demonstrated [21] that, after triacylglycerol hydrolysis of 10:0 and 12:0, FA are absorbed directly in the blood circulation without being incorporated in chylomicrons, thus may be more bioavailable and/or readily available sources of energy in the immature preterm digestive system that longer chain FA. Additionally, entering the cells these FA get into mitochondria without the assistance of a carnitine transporter [21], therefore sparing adenosine triphosphate (ATP) for other cellular process. Palmitic acid (16:0) was the most abundant saturated FA and accounted for approximately 60% of sum of SFA and it also represented the second most abundant FA in HM. Palmitic acid did not show any major temporal changes in either of the groups, suggesting minor variations amongst different populations, a phenomenon that has also been observed in other studies [36,37,38].

Amongst all FA characterized in this study, oleic acid (OA, 18:1 n-9) was the most abundant FA in both term and preterm groups. While it did not show any statistically significant difference between groups in colostrum and transitional milk, the content was higher in term mature milk. This observation is in line with findings of Rueda et al., [39] which not only agreed with the ranges of OA present in both term and preterm milk but also demonstrated that term HM contained higher proportions of OA in comparison to their preterm counterparts.

Linoleic acid (LA) was the most abundant n-6 FA in both term and preterm milk. Both groups showed slight increases in the concentrations from colostrum to mature milk. Not only is linoleic acid an essential fatty acid that is a required precursor for production of ARA, but also downstream products of ARA yields leukotrienes, prostaglandins and thromboxane that are physiologically active and provide diversified functions of signaling. The relative percentage of linoleic acid in our study agreed with previous reports of Luukkainen [37], Genzel-Boroviczény [38], Rueda [39] and Sabel [40]. However, Kovacs [36] reported to have 40 to 50% more LA in milk of both term and preterm infants. However, since n-6 FA can be modulated by maternal intakes, the groups can attribute the observation to differential intake. Alpha-linolenic acid (ALA), the precursor to EPA and DHA, represented between 40 and 60% of all n-3 FA characterized in this study for both term and preterm infants. In our study, colostrum of preterm HM contained statistically significant higher proportions of ALA than their term counterparts. However, this significance did not sustain over time and the relative percentages of ALA were comparable in both term and preterm groups for transitional and mature milk. The mature milk of other studies also reportedly has no differences between the groups [36,37]. A limitation of this study is that neither dietary intake, nor supplement intake was recorded preventing us from associating HM FA to these factors.

The concentration of DHA decreased over stages of lactation for both groups, preterm and term HM. This trend has been described by numerous studies reported in the literature [19,31,35,37,38,39]. However, consistency is not observed when comparing the concentrations of DHA in milk within term and preterm groups. In our study we did not observe significant differences, yet it has been reported by Kovács et al. [36] that there is significantly higher DHA in preterm milk than in term milk for first 21 day post-partum. On the other hand Rueda et al. [39], reported to have higher DHA in term milk over preterm milk for first week post-partum. Since DHA content of mothers milk is sensitive to maternal intake of food rich in sources of DHA [41], it may explain the differences observed in different studies. It also may be prudent to note that preterm offspring may benefit from fortifying mothers’ own milk in populations where dietary/supplementary intake of sources of DHA may be lower than ideal.

## 5. Conclusions

In summary, oleic, palmitic and linoleic acids were the most abundant FA across lactation and in both groups. Preterm colostrum contained significantly higher 8:0, 10:0, 12:0, sum medium chain fatty acids (MCFA), 18:3 n-3 FA compared to term counterparts. Preterm mature milk contained significantly higher 12:0, 14:0, 18:2 n-6, sum SFA, and sum MCFA. Preterm colostrum contained significantly lower 20:0, 20:3 n-6, 22:1 n-9 and 24:1 n-9. Preterm mature milk contained significantly lower total lipids, 16:1 n-7 and 18:1 n-9. We did not observe any significant differences between the preterm and term groups for DHA, ARA and EPA at any stage of lactation. Overall, preterm milk was higher for SFA with major contributions from MCFA and higher in 18:2 n-6. These observational differences need to be studied further for their implications on preterm developmental outcomes and on fortification strategies of either mothers’ own milk or donor human milk.

## Figures and Tables

**Table 1:** Maternal and infant characteristics of the study population.

Study Population	Preterm	Term	*p*-value
**Maternal**	***n* = 27**	***n* = 34**	
Age (years), mean ± SD	32.4 ± 5.6	31.2 ± 4.2	0.3173
Height (cm), mean ± SD	165.2 ± 7.1	166.8 ± 6.6	0.3601
Weight before pregnancy (kg), mean ± SD	62.1 ± 9.5	64.3 ± 12.0	0.4479
Weight at birth (kg), mean ± SD	70.3 ± 10.6	74.5 ± 11.3	0.1426
BMI before pregnancy (kg/m^2^), mean ± SD	22.8 ± 3.4	23.2 ± 4.9	0.6990
BMI at birth (kg/m^2^), mean ± SD	25.8 ± 3.7	26.9 ± 4.7	0.3141
Caesarean delivery, %	63.0	23.5	0.0019
**Infant**	***n* = 33**	***n* = 34**	
Gestational age at birth (weeks), mean ± SD	30.8 ± 1.4	39.5 ± 1.0	<0.0001
Males, %	54.5	52.9	0.8952
Twins, %	36.4	0.0	0.0001
Height (cm), mean ± SD	40.4 ± 3.2	49.4 ± 1.7	<0.0001
Weight (g), mean ± SD	1421.4 ± 372.8	3277.6 ± 353.6	<0.0001
Head circumference (cm), mean ± SD	27.8 ± 2.1	34.4 ± 1.5	<0.0001

**Table 2:** Fatty acid (FA) composition of human milk (HM) expressed by mothers who delivered either preterm or term infant(s).

Fatty Acids	Colostrum		Transitional Milk		Mature Milk	
	Preterm	Term	Preterm	Term	Preterm	Term
total lipids (g/100 mL)	2.40 (1.25)	1.70 (1.35)	3.05 (1.35)	3.10 (0.95)	3.10 ^c^ (1.40)	3.60 ^c^ (1.95)
8:0 (caprylic acid)	0.08 ^a^ (0.08)	0.03 ^a^ (0.05)	0.20 (0.09)	0.22 (0.04)	0.21 (0.08)	0.22 (0.06)
10:0 (capric acid)	0.60 ^a^ (0.44)	0.29 ^a^ (0.3)	1.58 (0.58)	1.64 (0.47)	1.48 (0.47)	1.46 (0.38)
12:0 (lauric acid)	3.14 ^a^ (2.22)	2.24 ^a^ (1.28)	6.76 (2.41)	6.33 (2.02)	5.91 ^c^ (2.44)	5.26 ^c^ (2.10)
14:0 (myristic acid)	6.20 (1.52)	5.83 (1.77)	7.86 (3.35)	7.62 (1.8)	7.36 ^c^ (2.9)	6.27 ^c^ (1.93)
16:0 (palmitic acid)	24.02 (1.97)	25.68 (2.83)	22.75 (4.86)	23.49 (3.11)	23.10 (3.46)	23.29 (3.31)
16:1 n-7 (palmitoleic acid)	2.34 (0.88)	2.18 (0.51)	1.96 (1.24)	2.34 (0.74)	2.17 ^c^ (0.82)	2.44 ^c^ (0.77)
18:0 (stearic acid)	6.30 (1.55)	6.79 (1.51)	6.27 (1.86)	6.23 (1.03)	7.03 (2.06)	6.75 (1.69)
18:1 n-9 (oleic acid)	37.64 (2.73)	39.36 (3.02)	34.62 (5.77)	35.85 (4.06)	35.22 ^c^ (5.16)	37.67 ^c^ (4.82)
18:1 n-7 (vaccenic acid)	2.62 (0.62)	2.67 (0.48)	2.06 (0.44)	2.13 (0.4)	1.83 ^c^ (0.45)	1.96 ^c^ (0.38)
18:1 trans fatty acids	0.70 (0.28)	0.75 (0.19)	0.68 (0.3)	0.75 (0.31)	0.71 ^c^ (0.42)	0.82 ^c^ (0.36)
18:2 n-6 (linoleic acid)	9.61 (2.19)	7.92 (1.17)	9.55 ^b^ (2.98)	8.70 ^b^ (2.23)	10.21 ^c^ (3.64)	9.35 ^c^ (2.90)
18:3 n-3 (α-linolenic acid)	0.77 ^a^ (0.25)	0.51 ^a^ (0.15)	0.72 (0.28)	0.67 (0.26)	0.75 (0.43)	0.74 (0.30)
18:3 n-6 (γ-linolenic acid)	0.03 (0.02)	0.03 (0.02)	0.05 (0.03)	0.08 (0.04)	0.09 (0.04)	0.10 (0.06)
20:0 (arachidic acid)	0.21 ^a^ (0.05)	0.27 ^a^ (0.09)	0.20 (0.06)	0.21 (0.04)	0.20 (0.07)	0.20 (0.04)
20:1 n-9 (eicosenoic acid)	0.76 (0.23)	0.99 (0.20)	0.60 ^b^ (0.17)	0.54 ^b^ (0.09)	0.47 (0.14)	0.45 (0.12)
20:2 n-6 (eicosadienoic acid)	0.52 (0.22)	0.58 (0.16)	0.42 ^b^ (0.12)	0.34 ^b^ (0.06)	0.29 (0.12)	0.26 (0.07)
20:3 n-6 (dihomo-γ-linolenic acid)	0.51 ^a^ (0.17)	0.66 ^a^ (0.35)	0.41 (0.10)	0.48 (0.18)	0.35 (0.12)	0.38 (0.13)
20:5 n-3 (EPA)	0.07 (0.07)	0.07 (0.03)	0.06 (0.05)	0.07 (0.03)	0.06 (0.04)	0.06 (0.04)
22:1 n-9 (erucic acid)	0.19 ^a^ (0.06)	0.25 ^a^ (0.06)	0.12 (0.06)	0.12 (0.02)	0.09 (0.03)	0.08 (0.03)
20:4 n-6 (ARA)	0.71 (0.36)	0.78 (0.32)	0.55 (0.19)	0.53 (0.11)	0.40 (0.12)	0.42 (0.12)
24:0 (lignoceric acid)	0.19 (0.05)	0.23 (0.1)	0.14 (0.05)	0.12 (0.04)	0.08 (0.04)	0.08 (0.03)
24:1 n-9 (nervonic acid)	0.30 ^a^ (0.09)	0.39 ^a^ (0.14)	0.13 (0.09)	0.13 (0.03)	0.07 (0.04)	0.07 (0.03)
22:6 n-3 (DHA)	0.61 (0.41)	0.64 (0.28)	0.35 (0.19)	0.42 (0.15)	0.27 (0.16)	0.28 (0.17)
Sum SFA	43.49 (7.03)	42.03 (2.84)	47.14(7.76)	46.08 (6.37)	45.88 ^c^ (7.45)	43.86 ^c^ (5.93)
Sum MUFA	44.59 (2.90)	46.51 (3.72)	40.65 (6.19)	42.12 (4.88)	40.44 ^c^ (5.6)	43.84 ^c^ (4.96)
Sum MCFA (< 14:0)	3.95 ^a^ (2.52)	2.51 ^a^ (1.65)	8.52 (2.67)	7.93 (2.66)	7.63 ^c^ (2.89)	6.94 ^c^ (2.38)
Sum PUFA	12.90 (2.95)	11.33 (1.95)	12.19 (3.05)	11.29 (2.03)	12.76 (4.00)	11.77 (3.43)
Sum PUFA n-3	1.48 (0.91)	1.16 (0.36)	1.24 (0.54)	1.15 (0.23)	1.15 (0.62)	1.11 (0.36)
Sum PUFA n-6	10.16 (2.68)	9.92 (1.66)	10.88 (2.96)	10.20 (2.22)	11.37 (3.82)	10.61 (3.17)
n-6 to n-3 ratio	7.29 (2.49)	8.67 (2.24)	8.68 (4.56)	8.58 (1.93)	9.72 (4.94)	9.58 (3.31)
ARA to DHA ratio	1.36 ^a^ (0.71)	1.40 ^a^ (0.53)	1.52 (0.59)	1.40 (0.41)	1.77 (1.15)	1.51 (0.68)

The data expressed in this table are medians (and interquartile range in parentheses) expressed as g/100 g FA except total lipids which is expressed in g/100 mL of human milk and ratios. Values within a row with a letter ^(a, b, c)^ indicate statistically significant differences (*p* < 0.05) between preterm and term HM for colostrum, transitional and mature milk, respectively. SFA—Saturated Fatty Acids, MUFA—Mono-Unsaturated Fatty Acids, PUFA—Poly-Unsaturated Fatty Acids, MCFA—Medium Chain Fatty Acids, ARA—Arachidonic Acid, DHA—Docosahexaenoic Acid, EPA—Eicosapentaenoic Acid.
